# Psychometric properties of the Turkish adaptation of the social issues advocacy scale-2 short form

**DOI:** 10.3389/fpsyg.2026.1776892

**Published:** 2026-06-24

**Authors:** Abdullah Mücahit Aslan, Fedai Kabadayi

**Affiliations:** 1 Department of Guidance and Counseling, Faculty of Education, Karamanoğlu Mehmetbey University, Karaman, Türkiye; 2 Department of Guidance and Counseling, Faculty of Education, Recep Tayyip Erdoğan University, Rize, Türkiye

**Keywords:** advocacy, counselor, reliability, scale adaptation, social issues advocacy, validity

## Abstract

**Background:**

Previous literature provides a limited number of measurement instruments for social issues advocacy. The present study focuses on the adaptation of the Social Issues Advocacy Scale-2 Short Form (SIAS-2 SF) into Turkish for counselor and counselor candidates and the evaluation of its psychometric properties.

**Methods:**

The research was performed in four phases. A total of 639 participants were employed, including 173 counselors and 466 counselor candidates (495 female, 144 male). The pilot study included 36 participants, the construct validity and reliability assessment phase involved 422 participants, the test-criterion validity and reliability assessment phase involved 148 participants, and the test–retest reliability phase involved 33 participants. The analysis was performed with JASP and JAMOVI software.

**Results:**

The results indicated that the Turkish version of the SIAS-2 SF, consisting of 38 items across eight dimensions, demonstrated acceptable model fit indices. Factor loadings ranged from 0.32 to 0.91. The Turkish version of the SIAS-2 SF showed satisfactory internal consistency in the first administration (Phase II: *ω* = 0.933, *α* = 0.933) and in the second administration (Phase III: *ω* = 0.934, *α* = 0.938), along with adequate test–retest reliability (*r* = 0.69). Social issues advocacy, social justice, and life satisfaction were found to be moderately and positively correlated.

**Conclusion:**

These findings suggest that the SIAS-2 SF is a useful and comprehensive measurement instrument for Turkish counselors and counselor candidates. The SIAS-2 SF can be utilized for pioneering research.

## 1 Introduction

Social justice advocacy has become increasingly important in improving the quality of life of disadvantaged people in different segments of society, combating discrimination and promoting social change. Social justice advocacy encompasses a range of actions, including raising individual awareness, addressing systemic inequalities and advocating for policy reform (Constantine et al., 2007). An analysis of the concept of social justice advocacy shows that it is a synthesis of two fundamental concepts. The first is the notion of social justice introduced by Constantine et al. (2007), which emphasizes the importance of justice and equity for individuals and groups who are marginalized. The second is Cohen’s (2001) definition of advocacy, which refers to actions aimed at changing the decision-making process in the public sphere. As a product of these two core concepts, social justice advocacy is the active effort to promote equity in social, political and economic systems, focusing on availability, resource allocation and civil rights (Fouad et al., 2006).

The field of counseling psychology has historically been one of the leading fields for social justice advocacy (Nilsson et al., 2011). In the early 1900s, Frank Parsons initiated this process by working with impoverished and unemployed youth in Boston, while Clifford Beers advocated better treatment and approaches to people with mental disorders. In the 1940s and 1950s, mental health practitioners supported the adjustment of war veterans to civilian society, and in the 1960s and 1970s they pushed for changes in education and core civil rights. From the 1900s to the present, the multicultural competence movement has positioned counselors as social justice advocates against inequality and discrimination.

In the discipline of counseling psychology, social justice advocacy involves not only providing individual-level support but also fostering change at a societal level (Nilsson et al., 2011). Traditional counseling approaches are predominantly individual-centered and may overlook systemic and environmental factors. However, given the effect of systemic factors such as impoverishment, discrimination, and educational inequalities on individuals’ mental health, it becomes essential for counselors to embrace and act upon social justice advocacy. Indeed, social injustices such as oppression, discrimination, and inequality are known to affect individuals’ psychological and physical health as well as their academic achievement (Yoshikawa et al., 2012).

Within the scope of social justice advocacy, counselors are anticipated to assist disadvantaged individuals and promote awareness in the fields of education and policy and engage in efforts to eliminate structural inequalities. In this context, the Advocacy Competencies framework formed by the American Counseling Association (ACA) outlines how counselors can effectively advocate at person, society, and public policy levels (Lewis et al., 2002). This model encourages counselors to adopt various roles, ranging from individual advocacy to systemic change. Despite the increasing attention to social justice and multiculturalism in counseling psychology literature, the lack of reliable and valid measurement instruments for assessing social justice advocacy remains a significant issue. Understanding and measuring how social justice advocacy develops among counselors is therefore of great importance.

Despite a growing body of empirical research on social justice advocacy, only a limited number of instruments have been developed to measure advocacy attitudes and actions. Nilsson et al. (2011) developed the Social Issues Advocacy Scale (SIAS) to assess individuals’ perspectives and behaviors related to social justice advocacy. The instrument consists of four sub-dimensions: Political and Social Advocacy, Confronting Discrimination, Political Awareness, and Social Issues Awareness. The SIAS has shown high internal consistency and has been used in various academic and professional settings (Nilsson et al., 2011). Over time, however, the SIAS was deemed insufficient to fully capture contemporary understandings of social justice and advocacy, leading to the improvement of SIAS-2 (Marszalek et al., 2017). SIAS-2 consists of eight sub-dimensions and aims to provide a more detailed assessment of social justice advocacy. However, its extensive 78-item structure has been criticized for being impractical to use. Consequently, a shorter and more applicable version, the SIAS-2 Short Form (SIAS-2 SF), has been developed (Nilsson et al., 2024). The SIAS-2 SF consists of 38 items and eight subscales, retaining the psychometric properties of the original instrument while improving its usability in assessment processes.

Several other instruments have also been developed to assess social justice advocacy. For instance, the Social Justice Advocacy Readiness Questionnaire, created by Chen-Hayes (2001), evaluates individuals’ awareness of and comfort with social justice issues. The Activism Orientation Scale (AOS) by Corning and Myers (2002) measures individuals’ tendencies toward social and political activism. Additionally, Dean (2009) developed the Social Justice Advocacy Scale, and Windsor et al. (2015) created the Social Work Advocacy Scale, both of which assess advocacy attitudes and actions across different professional groups. However, these instruments generally focus on specific professional groups or particular contexts of social justice advocacy rather than providing multidimensional measurements like SIAS-2 SF. Additionally, the high number of items in some of these instruments makes their use and evaluation challenging.

In recent years, advocacy in psychological counseling has become an important research topic in Türkiye. In order to measure social justice advocacy in Türkiye, various instrument adaptation studies have been performed. Kırlıoğlu and Tekin (2019) adapted the Social Justice Advocacy Scale (SJAS) developed by Dean (2009) into Turkish culture and demonstrated its validity and reliability. Similarly, Uz Baş et al. (2022) adapted the Self-Advocacy Scale for School Counselors into Turkish, showing its effectiveness in assessing school counselors’ self-advocacy skills.

The SIAS and SIAS-2 have been adapted into different cultural settings, and validity studies have been conducted. For example, Akin et al. (2013) adapted the SIAS into Turkish and examined its psychometric properties. A similar adaptation process was performed for the Korean culture (Lim, 2016). These studies indicated that the SIAS is applicable across different cultural settings. However, no validity and reliability studies have been implemented specifically for SIAS-2 SF among Turkish counselors and counselor candidates. Therefore, the aim of the present study is to adapt the SIAS-2 SF for Turkish counselors and counselor candidates and to assess its psychometric properties.

### 1.1 Rationale of the study

The adaptation of existing measurement instruments is widely recognized as an important methodological approach in cross-cultural psychological research, particularly when the construct under investigation has already been theoretically and empirically established (Hambleton and Patsula, 1999; Van de Vijver and Tanzer, 2004). Rather than developing a new scale for every cultural context, adaptation studies allow researchers to evaluate whether an existing construct maintains its conceptual and psychometric integrity across different cultural settings. Such an approach contributes to the accumulation of comparable scientific knowledge and enables cross-cultural comparisons within the international literature.

The decision to adapt the Social Issues Advocacy Scale-2 Short Form (SIAS-2 SF) instead of developing a new instrument was based on several theoretical and methodological considerations. First, the SIAS-2 SF is grounded in a comprehensive and well-established conceptualization of social justice advocacy within counseling psychology. The scale was developed through systematic empirical refinement processes and has demonstrated strong psychometric properties in measuring multidimensional aspects of advocacy-related attitudes, awareness, and behaviors (Nilsson et al., 2011; Marszalek et al., 2017; Nilsson et al., 2024). Therefore, the construct assessed by the SIAS-2 SF has already been clearly defined and operationalized in the literature, reducing the necessity of developing a completely new instrument.

Second, adaptation of an internationally recognized instrument provides important advantages for cross-cultural research. Using the Turkish adaptation of the SIAS-2 SF enables findings obtained from Turkish counselors and counselor candidates to be compared with findings from other cultural contexts. Such comparability is particularly important in counseling psychology because social justice advocacy is influenced by sociocultural factors while simultaneously reflecting universal professional competencies and values (Ratts et al., 2016). Developing a culturally specific scale would limit direct comparisons with the broader international literature and reduce the possibility of contributing to cumulative multicultural research.

The adaptation of the SIAS-2 SF is expected to contribute to the Turkish counseling literature in several important ways. Unlike existing advocacy-related instruments adapted into Turkish culture, the SIAS-2 SF does not focus solely on specific advocacy domains, profession-specific advocacy skills, or limited aspects of social justice engagement. Instead, the scale provides a comprehensive and multidimensional assessment of social justice advocacy by evaluating awareness, advocacy attitudes, discrimination confrontation, and sociopolitical engagement within a unified framework. In this respect, the SIAS-2 SF offers a broader conceptualization of advocacy compared to existing Turkish instruments, which generally assess narrower or context-specific dimensions of advocacy. In addition, the SIAS-2 SF reflects contemporary multicultural and social justice counseling perspectives that conceptualize counselors’ responsibilities not only at the individual level but also at systemic and societal levels. Therefore, the scale extends beyond traditional individual-centered advocacy approaches by incorporating social and structural dimensions of counseling practice. Another important strength of the SIAS-2 SF is its short-form structure, which enhances practicality and usability while preserving the multidimensional nature of the original instrument. Previous studies have indicated that lengthy instruments may create implementation difficulties and reduce participant response quality in applied research settings. In this respect, the SIAS-2 SF provides a balanced structure by combining psychometric strength with practical applicability in research, counselor education, and professional counseling settings.

Additionally, increasing sociocultural diversity and ongoing social justice concerns in Türkiye further increase the importance of assessing counselors’ advocacy competencies. Issues such as migration, socioeconomic inequality, gender-based discrimination, educational inequities, and refugee-related challenges have become increasingly visible within counseling and mental health contexts. In this respect, the availability of a comprehensive and psychometrically sound instrument for assessing social justice advocacy may contribute to both research and counselor training practices in Türkiye.

Considering these methodological, theoretical, and practical advantages, adaptation of the SIAS-2 SF was considered more appropriate than developing a new instrument for the Turkish culture. Accordingly, the present study aims to examine the validity and reliability of the Turkish form of the SIAS-2 SF among counselors and counselor candidates.

## 2 Methods and materials

### 2.1 Participants

The participants of the present study were employed in four phases via convenience sampling, with participants recruited from provinces covering all seven geographical regions of Türkiye. In each phase, participants were not included in the other phase and only participated in one phase.

#### 2.1.1 Phase I: pilot study

In the pilot study phase, 26 candidates and 10 counselors in total 36 participants were employed. The participants were 27 female and 9 males. The mean age of the participants was 23.19 (SD = 2.62, minimumage = 20, maximumage = 34). Five of the participants were members of the Turkish Psychological Counseling and Guidance Association.

#### 2.1.2 Phase II: construct validity and reliability assessment

For construct validity and reliability assessments, 422 participants were included in the study. 101 (%23.90) of the participants were counselors and 321 (%76.10) were counselor candidates. 95 (%22.50) of the participants were male and 327 (%77.50) were female. The mean age was 22.70 years (SD = 4.05, minimumage = 17, maximumage = 45). In terms of education level, 88 (%20.85) participants had a bachelor’s degree, 11 (%2.60) had a master’s degree, and 2 (%0.47) had a doctorate degree. Participants came from 66 different provinces of Türkiye (81 provinces in total). The majority of the participants (329 participants, %77.96) were not members of any professional organization. However, 81 (%19.20) participants were members of the Turkish Psychological Counseling and Guidance Association, 3 (%0.70) were members of the Red Crescent, 3 (%0.70) were members of professional unions, 3 (%0.70) were members of other organizations, 2 (%0.50) were members of the Council of Counselor Candidates and 1 (%0.20) was a member of the Green Crescent. When the grade levels of the prospective counselors were assessed, 105 (%32.80) of them were studying in the second grade, 104 (%32.50) in the third grade, 85 (%26.60) in the fourth grade and 26 (%8.10) in the first grade. In terms of work experience, 21 (%4.98) of the counselors had 10 years of experience or more, 20 (%4.74) had 3–10 years of experience, and 60 (%14.22) had 0–3 years of experience.

#### 2.1.3 Phase III: test-criterion validity and reliability assessment

In the test-criterion validity phase, a total of 148 participants were included in the study. Among them, 32 were male (21.62%) and 116 were female (78.38%). The participants consisted of 53 counselors (35.81%) and 95 counselor candidates (64.19%). Among the counselor candidates, 1 (1.05%) was in the first grade, 50 (52.63%) were in the second grade, and 42 (44.21%) were in the third grade. Among the counselors, 4 (7.55%) had a doctoral degree, 9 (16.98%) had a master’s degree, and 40 (75.47%) had a bachelor’s degree. The mean age of the participants was 23.17 years (SD = 4.15, minimum_age_ = 18, maximum_age_ = 40). Regarding professional memberships, 108 participants (72.97%) were not affiliated with any professional organization, while 32 (21.62%) were members of the Turkish Psychological Counseling and Guidance Association, and 8 (5.41%) were affiliated with other organizations. Participants resided in 44 different provinces across Türkiye.

#### 2.1.4 Phase IV: test–retest reliability

A total of 33 participants, comprising 25 women and 8 men, were included in the test–retest phase. Among the participants, 24 were counselor candidates, and 9 were practicing counselors. The mean age of the participants was 23.03 years (SD = 2.44, minimumage = 20, maximumage = 34). Regarding professional memberships, 28 participants were not affiliated with any professional organizations, while 5 were members of the Turkish Psychological Counseling and Guidance Association. Participants resided in 17 different provinces across Türkiye.

### 2.2 Instruments

We utilized three measurement instruments and a personal information form.

#### 2.2.1 Short form of the social issues advocacy scale–2

The measurement instrument aims to measure social justice advocacy (Nilsson et al., 2024). The measurement instrument consists of eight dimensions: relationship building, political and social advocacy, sense of community responsibility, social issue awareness, political awareness, willingness to challenge and confront, social justice self-efficacy, confront discrimination. The measurement instrument consists of 38 items and is a 5-point Likert type. The item factor loadings of the measurement instrument are between 0.54 and 0.88. The Cronbach’s (*α*) coefficient of the instrument was between 0.94 (subscale (*α*) 0.74–0.87) (Nilsson et al., 2024). In the present study we calculated Cronbach’s (*α*) and McDonald’s (*ω*) coefficients. The values for Cronbach’s (*α*) and McDonald’s (*ω*) were 0.933 in the second phase of the study, while in the third phase, which focused on criterion validity, the values were 0.938 for Cronbach’s (*α*) and 0.934 for McDonald’s (*ω*).

#### 2.2.2 Social justice scale

The measurement instrument was developed in a 4-factor structure including attitude, behaviour, norm, and intention and aims to assess the level of importance and value given to activities related to social justice (Torres-Harding et al., 2012). Turkish adaptation of the measurement instrument was provided by Cirik (2015). The measurement instrument consists of twenty-four items and is a 7-point Likert type. The instrument was found to have satisfactory structural properties (χ^2^/df = 2.72, TLI = 0.97, CFI = 0.97, RMSEA = 0.05, SRMR = 0.04). The item factor loadings of the measurement instrument are between 0.55 and 0.91. The Cronbach’s (*α*) coefficient of the instrument was between 0.82 and 0.95 (Cirik, 2015). In the phase III, we calculated the reliability coefficients Cronbach’s (α) and McDonald’s (*ω*), which were found to be 0.957 and 0.956, respectively.

#### 2.2.3 Satisfaction with life scale

The Satisfaction with Life Scale is a unidimensional measurement instrument developed by Diener et al. (1985). Turkish adaptation of the measurement instrument was provided by Dağlı and Baysal (2016). The measurement instrument consists of five items and is a 5-point Likert type. The measurement instrument showed strong structural properties (TLI = 1.00, CFI = 1.00, GFI = 0.99, RMSEA = 0.03, SRMR = 0.02). The item factor loadings of the instrument were between 0.73 and 0.89. The Cronbach’s (α) coefficient of the instrument was 0.88 (Dağlı and Baysal, 2016). In the phase III, we calculated the reliability coefficients Cronbach’s (α) and McDonald’s (ω), which were found to be 0.867 and 0.877, respectively.

#### 2.2.4 Personal information form

The questionnaire includes several questions regarding age, gender, education level, city of residence, professional organizations of which the respondent is a member, employment status and experience.

### 2.3 Adaptation process and procedure

A number of procedures were followed in the adaptation process. Accordingly, the original version of the measurement instrument was translated into Turkish by four experts in the discipline and one measurement and evaluation researcher, all of whom hold a PhD degree and are independent researchers from different universities. Then, the translations of each item were gathered together and two experts, also holding PhD degrees, decided on the most appropriate items for Turkish translation. Minor corrections were also made to the Turkish measurement instrument by a Turkish language expert, who holds a PhD, in order to make it fluent. Then, the Turkish form was back-translated into the original language form by an academic from the department of English language, a native-level bilingual independent researcher with a PhD and compared with the original measurement instrument and the semantic equivalence of all items with the original measurement instrument was ensured.

### 2.4 Procedure and data collection

Permissions were requested for the measurement instruments to be utilized before the research data were collected. Then, ethics committee approval was obtained from the Social and Human Sciences Ethics Committee of Recep Tayyip Erdoğan University (Date: 28.10.2024, Decision Number: 2024/368), and research permission was granted (Date: 30.10.2024). After the translation procedure was completed, the participants were included in the study. The main inclusion criteria were that the participants were students or graduates of the psychological counseling and guidance department and agreed to participate voluntarily. Data were collected over a period of approximately 3 months. Students participated in the study with their smartphones and were directed to Google Forms via QR code in the classroom environment. Graduates and employee counselors were contacted via e-mail and invited to participate in the study. All participants were asked to complete a voluntary consent form before participating in the study.

### 2.5 Data analysis

First, we preliminarily analysed the we used JASP and JAMOVI software to analyze the research data. First, we preliminarily analysed the data and verified that there were no missing data. Then, we analysed the item statistics and assessed the mean, standard deviation, minimum and maximum scores, skewness and kurtosis values of the instrument. In our study, we defined certain criteria to evaluate the results of confirmatory factor analysis (CFA). We used a minimum score of 0.90 for CFI and TLI values and a maximum score of 0.08 for SRMR and RMSEA (Hu and Bentler, 1999). These thresholds were used as the main criteria for assessing the model fit of the instrument. In addition, we expected the factor loading for each item to be at least 0.32 (Comrey and Lee, 1992). To assess the internal consistency of the measurement instrument, we calculated Cronbach’s (*α*) and McDonald’s (*ω*) coefficients, aiming for higher scores to indicate stronger internal reliability. Reliability coefficients of 0.70 or higher were regarded as indicative of acceptable internal consistency, based on commonly accepted criteria Nunnally and Bernstein, 1994. Network analysis has become an increasingly common method in the social sciences. Beyond correlation analyses, network analysis provides nodes and edges in the network plane. Nodes construct variables and edges show relationships (Love et al., 2019). In this study, edges between nodes were defined using a correlation estimator, and the threshold value was set to 0, meaning that all correlations were included in the network. In network analysis, centrality measures are important indicators that assess the connections of nodes in the network. Betweenness indicates how often a node uses the shortest paths between other nodes. Closeness measures the total inverse of a node’s distances to other nodes. Strength shows the total link strength of a node and emphasizes the central position of this node (Wagenmakers et al., 2020). The Fruchterman-Reingold layout algorithm was employed for network visualization. In the third phase, Pearson correlation analysis was performed to explore the relationships between social issues advocacy, social justice, and life satisfaction. The statistical significance of the correlations was tested at the *p* < 0.001 level, and the magnitude of the correlation coefficients was interpreted based on Cohen’s criteria (Cohen, 1988). Finally, in the fourth phase, we aimed to provide results showing that the psychometric properties of the measurement instrument are strong and consistent by conducting a test–retest study.

## 3 Findings

### 3.1 Phase I: pilot study

In the pilot study, the responses to the measurement instrument were analysed in terms of item statistics and the skewness and kurtosis values of each item were checked (see Table 1). The item statistics provided preliminary information about the items for the next phase.

**Table 1:** Item statistics.

Items	Mean	SD	Skewness	Kurtosis	Minimum	Maximum
I1	4.389	0.599	−0.389	−0.617	3.00	5.00
I2	4.500	0.609	−0.802	−0.264	3.00	5.00
I3	4.056	0.715	−0.082	−0.953	3.00	5.00
I4	3.861	0.899	−0.213	−0.845	2.00	5.00
I5	3.472	0.810	−0.417	−0.415	2.00	5.00
I6	3.000	1.146	−0.120	−0.723	1.00	5.00
I7	3.028	1.207	−0.262	−0.901	1.00	5.00
I8	3.611	1.022	−0.999	0.866	1.00	5.00
I9	2.639	1.246	0.087	−1.079	1.00	5.00
I10	4.583	0.649	−1.322	0.663	3.00	5.00
I11	4.194	0.668	−0.850	2.148	2.00	5.00
I12	4.444	0.652	−1.419	3.863	2.00	5.00
I13	4.361	0.762	−1.143	1.220	2.00	5.00
I14	4.278	0.741	−0.951	1.121	2.00	5.00
I15	3.278	1.031	0.226	−1.073	2.00	5.00
I16	3.806	1.009	−0.823	0.491	1.00	5.00
I17	4.333	0.717	−1.094	1.769	2.00	5.00
I18	4.389	0.645	−0.575	−0.549	3.00	5.00
I19	3.056	1.170	−0.227	−0.852	1.00	5.00
I20	4.361	0.723	−1.162	1.814	2.00	5.00
I21	3.472	0.910	−0.154	0.471	1.00	5.00
I22	4.028	0.774	−0.440	−0.096	2.00	5.00
I23	4.250	0.841	−1.126	1.046	2.00	5.00
I24	4.167	0.811	−0.663	−0.140	2.00	5.00
I25	4.528	0.609	−0.916	−0.078	3.00	5.00
I26	3.778	0.929	−0.884	1.188	1.00	5.00
I27	4.417	0.604	−0.487	−0.582	3.00	5.00
I28	3.972	1.055	−0.869	−0.346	2.00	5.00
I29	4.306	0.822	−0.964	0.225	2.00	5.00
I30	3.361	0.961	0.215	−0.807	2.00	5.00
I31	3.028	1.230	−0.153	−0.880	1.00	5.00
I32	4.111	0.820	−1.529	4.711	1.00	5.00
I33	4.278	0.615	−0.233	−0.506	3.00	5.00
I34	3.778	1.017	−0.731	0.289	1.00	5.00
I35	4.000	0.986	−1.517	3.123	1.00	5.00
I36	4.056	0.984	−0.878	−0.109	2.00	5.00
I37	4.000	0.986	−1.137	1.397	1.00	5.00
I38	4.500	0.655	−0.970	−0.090	3.00	5.00

### 3.2 Phase II: construct validity and reliability assessment

Construct validity studies conducted using CFA showed that the model fit was ensured (χ^2^/sd = 2.07, CFI = 0.91, TLI = 0.90, SRMR = 0.055, RMSEA = 0.050). Item factor loadings in the instrument were satisfactory. In literature, item factor loadings are considered to be minimum 0.32. Item factor loadings in the present study were between 0.32 and 0.91 (see Table 2).

**Table 2:** Factor loadings.

Items	D1	D2	D3	D4	D5	D6	D7	D8
I3	0.39							
I14	0.56							
I22	0.59							
I27	0.57							
I33	0.63							
I6		0.69						
I7		0.87						
I9		0.64						
I19		0.84						
I31		0.91						
I34		0.77						
I5			0.42					
I13			0.50					
I16			0.55					
I32			0.54					
I35			0.49					
I1				0.32				
I2				0.39				
I18				0.62				
I20				0.55				
I23				0.65				
I4					0.49			
I11					0.56			
I28					0.57			
I36					0.56			
I8						0.67		
I17						0.52		
I24						0.57		
I25						0.59		
I37						0.60		
I15							0.70	
I21							0.79	
I26							0.72	
I30							0.91	
I10								0.61
I12								0.64
I29								0.74
I38								0.59

D1 = relationship building, D2 = political and social advocacy, D3 = sense of community responsibility, D4 = social issue awareness, D5 = political awareness, D6 = willingness to challenge and confront, D7 = social justice self-efficacy, D8 = confront discrimination.

The reliability scores of the measurement instrument were found to be satisfactory, and the correlations between the dimensions were also examined (see Table 3).

**Table 3:** Correlations and reliability.

Variable	D1	D2	D3	D4	D5	D6	D7	D8	SIAS-2 SF
D1	–								
D2	0.493**	–							
D3	0.723**	0.460**	–						
D4	0.434**	0.166**	0.375**	–					
D5	0.545**	0.512**	0.487**	0.388**	–				
D6	0.667**	0.561**	0.618**	0.374**	0.547**	–			
D7	0.469**	0.491**	0.450**	0.117*	0.401**	0.574**	–		
D8	0.506**	0.276**	0.534**	0.319**	0.367**	0.692**	0.394**	–	
Phase II ω	0.784	0.826	0.725	0.806	0.691	0.762	0.853	0.843	0.933
Phase II α	0.782	0.821	0.730	0.799	0.663	0.760	0.847	0.842	0.933
Phase III ω	0.781	0.790	0.752	0.845	0.732	0.770	0.841	0.877	0.934
Phase II α	0.779	0.788	0.752	0.838	0.712	0.793	0.831	0.867	0.938

*p < 0.05, **p < 0.001; D1 = relationship building, D2 = political and social advocacy, D3 = sense of community responsibility, D4 = social issue awareness, D5 = political awareness, D6 = willingness to challenge and confront, D7 = social justice self-efficacy, D8 = confront discrimination.

**Figure 1:**
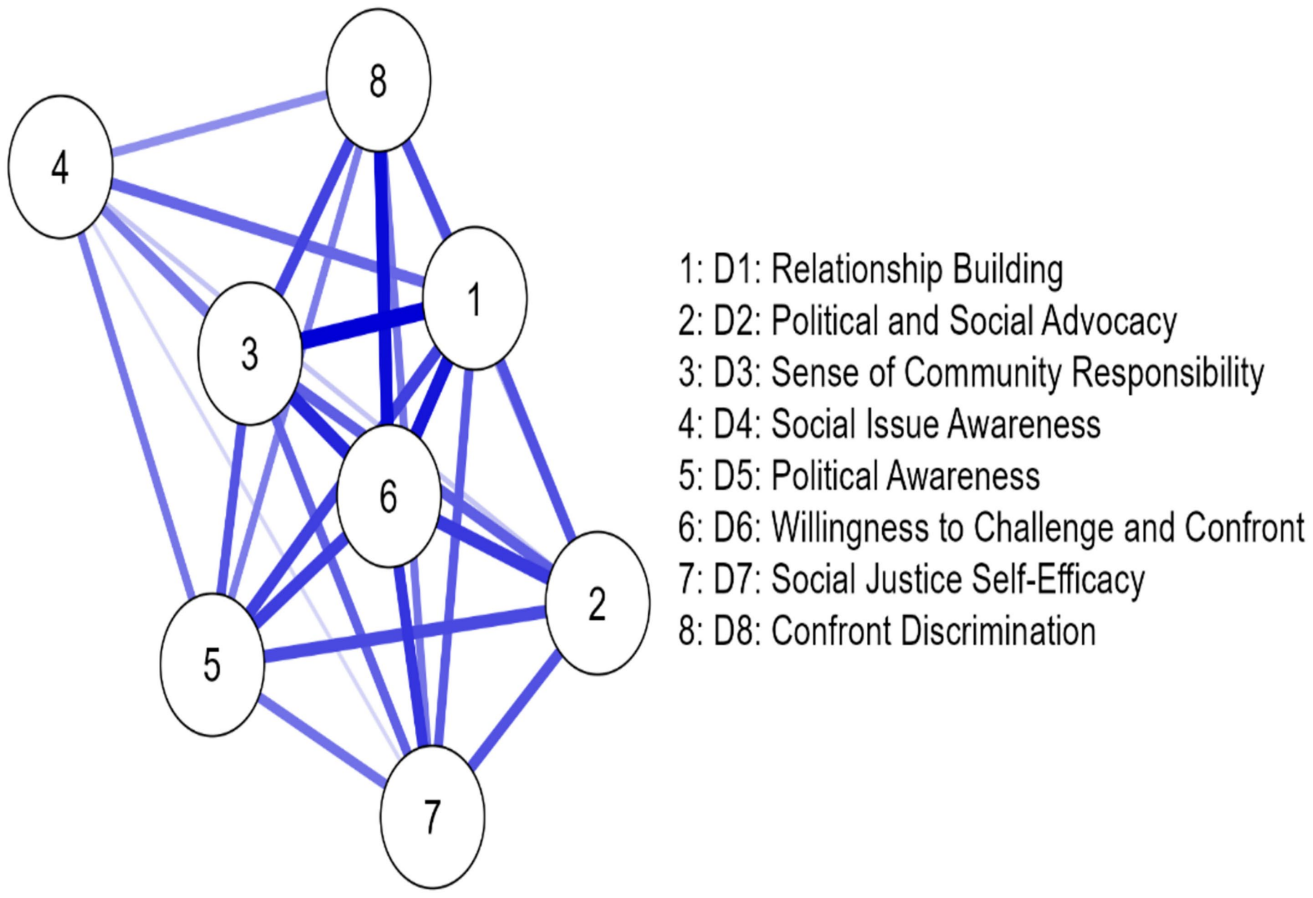
Network analysis of dimensions.

As expected, positive and significant correlations were observed among all dimensions. Moreover, network analysis was performed to provide further insights beyond the significant positive correlations between the dimensions (see Figure 1). The results showed that the dimension “willingness to challenge and confront” had the highest betweenness (2.18), closeness (1.37), and strength (1.33) scores. In Figure 1, the edges represent the relationships between the nodes (dimensions). Edge thickness and color intensity are proportional to the strength of the association; thicker and darker edges indicate stronger associations, whereas thinner and lighter edges indicate weaker associations between the nodes.

### 3.3 Phase III: test-criterion validity and reliability assessment

We assessed the relationships between the social issues advocacy, social justice, and life satisfaction in the third phase (see Table 4). The results indicated moderate, positive, and significant correlations between the social issues advocacy (*r* = 0.67, *p* < 0.001) and social justice and life satisfaction (*r* = 0.34, *p* < 0.001). These findings provide evidence for the criterion validity of the measurement instrument. Moreover, reliability scores of the measurement instrument were calculated and found to be satisfactory, with a second calculation conducted to ensure consistency (See Table 3).

**Table 4:** Test criterion validity.

Variable	M	SD	(1)	(2)	(3)
1. Social issues advocacy	145.39	18.72	–		
2. Social justice	137.53	20.18	0.67*	–	
3. Life satisfaction	16.05	3.92	0.34*	0.36*	–

*p < 0.001.

### 3.4 Phase IV: test–retest reliability

During the test–retest reliability phase, the instrument was implemented with a 6-week interval. Pearson correlation results indicated a satisfactory of reliability between the two administrations (*r* = 0.69, *p* < 0.001).

## 4 Discussion

The present study focused on the reliability and validity of the SIAS-2 SF in counselors and candidates. The measurement instrument evaluates social justice advocacy in a multidimensional framework, comprising 38 items distributed across 8 distinct dimensions. The results indicate that the measurement instrument has strong psychometric properties, demonstrating high reliability and strong construct validity.

The factor structure of the SIAS-2 SF was confirmed by CFA, network analysis and criterion validity. First, the SIAS-2 SF, consisting of 38 items and eight dimensions, showed an acceptable level of fit indices, confirming the factor structure. These results were consistent with the original form as previously reported in the literature (Nilsson et al., 2024). Factor loadings in psychometric research are generally considered to be above 0.30 or 0.32, as widely accepted in the literature (Carpenter, 2018; Seçer, 2015). In the present study, the factor loadings of the SIAS-2 SF ranged from 0.32 to 0.91, providing strong evidence that all items had considerable loadings. Furthermore, the factor loadings of the original instrument ranged from 0.54 and 0.88 (Nilsson et al., 2024), indicating that the SIAS-2 SF maintained a structure consistent with the original form. These results suggest that the Turkish version of the SIAS-2 SF effectively preserves the psychometric integrity of the original instrument while providing a concise measurement instrument.

Secondly, as expected, numerous significant positive correlations were identified between the subdimensions of Turkish version of the SIAS-2 SF. These correlation results suggest that the SIAS-2 SF consists of coherent and logically structured dimensions. Compared to the original study (Nilsson et al., 2024), the correlations between the dimensions followed a similar pattern, confirming the expected positive relationships between all subdimensions. Thirdly, previous studies on the original measurement instrument did not provide additional evidence for the factor structure of the SIAS-2 SF using network analysis. Therefore, the present study extends previous research by investigating the factor structure of the SIAS-2 SF within a network framework. Network analysis was performed to provide deeper insights into the dimensional structure and revealed that the Willingness to Challenge and Confront dimension had the highest centrality in terms of betweenness, closeness and strength. In recent psychological network literature, high strength identifies a node as a local hub that shares strong direct associations with many other nodes, whereas high betweenness indicates a bridging function that connects different parts of the network, and high closeness suggests the node can quickly affect or be affected by changes across the entire system (Wagenmakers et al., 2020; Borsboom & Cramer, 2013; Epskamp et al, 2018; Love et al., 2019). The co-occurrence of these three centrality indices in the same dimension implies that Willingness to Challenge and Confront is not merely an isolated element but rather a structurally integral component of the social justice advocacy network measured by the SIAS-2 SF. From a network perspective on psychological constructs such a central node may play a key role in organizing and activating related dimensions. The present study provides further insight into the factor structure of the instrument by incorporating the results of network analysis, thereby expanding on the structure previously identified in the literature.

Finally, in terms of criterion validity, significant moderate positive correlations with *Social Justice* and *Satisfaction with Life* provide further evidence supporting the validity of the SIAS-2 SF. In the previous original study, SIAS-2 SF showed significant positive relationships with *Social Justice Self-Efficacy*, *Social Justice Commitment*, *Political Engagement Index*, and *Cultural Openness*, while demonstrating a significant negative relationship with *Cultural Dominance* (Nilsson et al., 2024). Although the present findings have certain limitations that may restrict direct comparisons of patterns across different language versions of the instrument, the original study provides evidence that the Turkish version of the SIAS-2 SF has strong construct validity.

Reliability analyses yielded satisfactory results, with Cronbach’s alpha and McDonald’s omega coefficients both calculated as 0.933 in Phase I and 0.938 and 0.934, respectively, in Phase II. Additionally, the test–retest correlation was found to be 0.69, indicating sufficient reliability. In the original study, the total Cronbach’s alpha was reported as 0.94, with subscale alphas ranging from 0.74 to 0.87, while the test–retest correlation was 0.84 (Nilsson et al., 2024). These findings demonstrate that the Turkish version of the SIAS-2 SF provides adequate and satisfactory reliability evidence.

When compared to widely employed instruments in the literature, such as the Social Justice Scale (SJS) (Torres-Harding et al., 2012), the Activism Orientation Scale (AOS) (Corning and Myers, 2002), the Social Issues Questionnaire (SIQ) (Miller et al., 2009), the Social Justice Advocacy Scale (SJAS) (Dean, 2009), and the School Counselor Self-Advocacy Questionnaire (SCSAQ) (Clemens et al., 2011), the SIAS-2 SF has several critical advantages. Comparing the instruments first in terms of their purpose, the SIAS-2 SF is a measure of an individual’s level of social justice advocacy. This instrument aims to assess individuals’ awareness of social issues, their political and social advocacy tendencies, and their ability to confront and challenge discrimination. In contrast, the SJS assesses individuals’ attitudes, norms, intentions and behaviors related to social justice (Torres-Harding et al., 2012), while the AOS focuses on general activism orientation (Corning and Myers, 2002). The SIQ assesses interest in social justice issues (Miller et al., 2009), the SJAS explores levels of social service advocacy (Dean, 2009), and the SCSAQ measures school counselors’ ability to advocate for their professional roles and rights (Clemens et al., 2011). In contrast to these instruments, the SIAS-2 SF not only measures social justice awareness, but also assesses individuals’ active engagement in advocacy processes. In particular, the inclusion of unique dimensions such as “social justice self-efficacy” and “confronting discrimination” distinguishes the SIAS-2 SF from other instruments.

In terms of target populations and contexts, the SIAS-2 SF was originally developed for professionals and trainees in psychological counseling, education and social services. In this study the SIAS-2 SF was adapted for counselors and counselor candidates in Türkiye. In contrast, the SJS and SIQ are more broadly applicable to the general adult population and university students (Miller et al., 2009; Torres-Harding et al., 2012), while the AOS is designed for people involved in political activism and social movements (Corning and Myers, 2002). Although the SJAS was initially developed for counselors, its Turkish adaptation by Kırlıoğlu and Tekin (2019) demonstrated its applicability to social workers, social policy researchers, and the general population. The SCSAQ was designed specifically for school counselors to assess their professional self-advocacy skills (Clemens et al., 2011). In light of these considerations, the adapted SIAS-2 SF emerges in this study as a critical instrument for enhancing the professional development of counselors and counselor candidates. Unlike the AOS, SJS, SIQ and SJAS, which are designed for broader populations, the SIAS-2 SF is closely related to the SCSAQ in terms of its intended audience and context of implementation. However, the SIAS-2 SF provides a more in-depth and comprehensive assessment of social justice advocacy, addressing a critical gap in the field and reinforcing the role of counselors as key agents of social change.

In terms of item count, dimensions, and practicality, the SIAS-2 SF consists of 38 items and encompasses eight dimensions: relationship building, political and social advocacy, sense of community responsibility, social issue awareness, political awareness, willingness to challenge and confront, social justice self-efficacy, and confront discrimination. In comparison, the AOS consists of two dimensions (conventional activism and high-risk activism) with 35 items (Corning and Myers, 2002), while the SIQ consists of 52 items and six dimensions: social justice self-efficacy, social justice outcome expectations, social justice interest, social justice commitment, social justice supports, and social justice barriers (Miller et al., 2009). The SJS consists of 24 items across four dimensions (attitudes towards social justice, perceived behavioural control, subjective norms and behavioral intentions) (Torres-Harding et al., 2012). The SJAS has four dimensions (collaborative action, social/political advocacy, client empowerment, and client/community advocacy), and the SCSAQ is a single-factor instrument with nine items (Dean, 2009). With its multidimensional framework, balanced number of items and comprehensive coverage of social justice advocacy, the SIAS-2 SF stands out as a more holistic and robust assessment instrument compared to other instruments. In particular, the SIAS-2 SF’s emphasis on direct engagement in advocacy—encompassing key dimensions such as social justice self-efficacy and confronting discrimination—enables a deeper and more nuanced analysis of advocacy-related competencies. While some alternative measures may have fewer items, their comparatively narrower conceptual scope may limit their applicability in real-world settings, potentially limiting their effectiveness in capturing the complexity of social justice advocacy.

From a psychometric perspective, the SIAS-2 SF adaptation process employed CFA, Network Analysis, Criterion Validity, Internal Consistency (Cronbach’s *α* and McDonald’s *ω*), and Test–Retest Analysis. The results of these analyses have been detailed above. In contrast, the development of the AOS utilized Principal Component Analysis, Criterion Validity, and Internal Consistency (Cronbach’s α), but did not report CFA or test–retest reliability. The total variance explained by the first and second factors in the AOS was 83.4 and 16.6%, respectively, with internal consistency coefficients ranging from 0.87 to 0.96. In the development of SIQ, EFA was not used, CFA was used, criterion validity was examined with similar scales and internal consistency coefficients (Cronbach’s α) ranging from 0.79 to 0.94 were reported. Convergent and divergent validity evidence is not sufficient. In the case of the SJS, EFA was not used, but it is supported by CFA evidence. Strong evidence for convergent and divergent validity is available, with internal consistency (Cronbach’s α) reported between 0.82 and 0.95. However, insufficient information is provided regarding the evaluation of the factor structure, and test–retest reliability data have not been reported. The SJAS relied on Principal Component Analysis, Criterion Validity, and Internal Consistency (Cronbach’s α), but did not report CFA or test–retest reliability, explaining 41.82% of the total variance. The SCSAQ was evaluated using EFA, CFA, Criterion Validity, and Internal Consistency (Cronbach’s α), demonstrating a unidimensional structure accounting for 80% of the variance, with internal consistency coefficients between 0.84 and 0.87. When comparing the scales overall, it is generally observed that they meet validity and reliability criteria. However, except for the adapted SIAS-2 SF, the other scales typically lack CFA, test–retest reliability, and criterion validity analyses. In this regard, our current study utilizes advanced techniques, such as network analysis, in addition to basic psychometric analyses in the adaptation of the SIAS-2 SF, distinguishing it from other scales. Thus, it can be said that, not only in terms of validity and reliability, but also in modeling the relationships between the scale’s dimensions, it possesses a robust psychometric foundation.

## 5 Conclusion

In the present study, validity and reliability analyses of the Turkish adaptation of the SIAS-2 SF were conducted and the instrument was found to have a psychometrically strong structure. CFA confirmed the eight-dimensional structure with acceptable model fit indices. Criterion validity analyses showed significant positive correlations with instruments of social justice and life satisfaction. High internal consistency coefficients (Cronbach’s alpha and McDonald’s *ω*) also indicated satisfactory. The use of multiple analytical approaches, including network analysis, provided a deeper understanding of the dimensional relationships, highlighting ‘willingness to challenge and confront’ as a central factor. As a comprehensive instrument assessing both cognitive and behavioural components of social justice advocacy, the SIAS-2 SF is valuable for assessing individuals’ awareness and advocacy skills. In addition, this study represents the first cross-cultural adaptation of the SIAS-2 SF, contributing to its applicability in diverse settings.

### 5.1 Limitations

This study has several limitations that should be considered when interpreting the findings. The sample was limited to counselors and counselor candidates in Türkiye. Therefore, the findings may not be generalizable to other professional groups or different cultural contexts.

Another limitation concerns the online data collection process. Since the data were collected through online forms, participant responses may have been influenced by the digital survey environment. In addition, because the study relied on self-report measures, the findings may be subject to self-report bias and social desirability bias. Given the socially sensitive nature of social justice advocacy, some participants may have responded in ways that reflect socially desirable attitudes rather than their actual advocacy-related behaviors.

Furthermore, the cross-sectional nature of the study limited the examination of longitudinal stability and changes in social justice advocacy over time. Although the present study provided comprehensive psychometric evidence through CFA, criterion validity, reliability analyses, and network analysis, further psychometric evidence regarding cross-cultural invariance and predictive validity is still needed to strengthen the applicability of the Turkish form of the SIAS-2 SF across different contexts.

The sample was limited to counselors and counselor candidates in Türkiye, and future research should include different professional groups to increase generalizability. In addition, as the data were collected online, participants’ responses may have been influenced by the digital survey format. Future studies should consider face-to-face data collection to assess applicability more comprehensively. Further cross-cultural validation studies are recommended to examine the applicability of the instrument in different cultural contexts. Longitudinal studies exploring the relationship between social justice advocacy and professional practice would also provide valuable insights. Finally, structural equation modeling could further enhance the understanding of the structural properties of the Turkish version of the SIAS-2 SF in future research.

### 5.2 Recommendations

*Researchers*. The findings of the present study provide several recommendations for future research. Future studies may examine the psychometric properties of the Turkish form of the SIAS-2 SF among different professional groups working in helping professions, such as social workers, psychologists, and educators. Longitudinal and cross-cultural studies may also contribute to understanding how social justice advocacy develops over time and across different sociocultural contexts. In addition, future research may investigate the predictive role of social justice advocacy in relation to professional competence, multicultural counseling competence, psychological well-being, and counselor effectiveness.

Additionally, future studies may focus on exploring the relationships of the SIAS-2 SF with a broader range of relevant constructs. Relevant constructs may include social support, activism tendencies (e.g., conventional and high-risk activism), and forms of political participation such as voting, contacting elected officials, and collective action. These associations may help clarify how the construct measured by the SIAS-2 SF is embedded within broader psychosocial and behavioral processes. In addition, weak or negligible relationships with unrelated constructs, such as social desirability, would further contribute to understanding the specificity of the scale.

*Practitioners*. The present findings also provide important implications for counseling practitioners and counselor educators. The Turkish form of the SIAS-2 SF may help practicing counselors evaluate their own advocacy-related strengths and areas requiring further professional development. In particular, the multidimensional structure of the instrument may support counselors in recognizing their levels of discrimination awareness, sociopolitical engagement, willingness to confront injustice, and social justice self-efficacy within counseling practice. In school, university, and community mental health settings, counselors may use the findings obtained from the SIAS-2 SF to better understand how social and systemic factors influence clients’ well-being. The instrument may also support practitioners in developing more culturally responsive and socially sensitive counseling approaches when working with disadvantaged and marginalized populations. Furthermore, the SIAS-2 SF may contribute to supervision and in-service training processes by increasing awareness of advocacy-related competencies among counselors and counselor candidates.

*Policymakers*. The findings may provide implications for policymakers and institutions involved in counselor education and mental health services. Given the increasing visibility of migration, socioeconomic inequality, educational inequities, gender-based discrimination, and refugee-related challenges in Türkiye, policymakers may consider integrating social justice advocacy competencies into counselor education standards, training programs, and professional development initiatives. In this respect, the SIAS-2 SF may serve as a useful assessment instrument for evaluating advocacy-related competencies and supporting policies that promote inclusive and socially responsive counseling practices.

## Data Availability

The raw data supporting the conclusions of this article will be made available by the authors, without undue reservation.
